# THE ASSOCIATION BETWEEN COGNITION AND UPPER EXTREMITY MOTOR REACTION TIME IN OLDER ADULTS: A NARRATIVE REVIEW

**DOI:** 10.1093/geroni/igad104.2136

**Published:** 2023-12-21

**Authors:** Alexandria Jones, Natalie Weaver, Mardon So, Abbis Jaffri, Rosalind Heckman

**Affiliations:** Creighton University, Omaha, Nebraska, United States; Creighton University, Omaha, Nebraska, United States; Creighton University, Omaha, Nebraska, United States; Creighton University, Omaha, Nebraska, United States; Creighton University, Omaha, Nebraska, United States

## Abstract

Response timing is essential to optimal sensorimotor control across the lifespan. While it is broadly assumed that reaction time increases as cognition declines with age, it is unclear if this assumption is supported by the literature. The purpose of this narrative review was to determine the association between cognition and upper extremity reaction time in older adults. Cognitive domains of sensation and perception, motor construction, perceptual motor function, executive function, attention, learning and memory, and language were considered. We conducted a systematic search using Scopus database. The search strategy was designed to meet four inclusion criteria: 1) community-dwelling adults >60 years, 2) upper extremity motor task, 3) at least one cognitive assessment, 4) simple reaction time measure. 1154 articles were screened. Two articles met the full inclusion criteria, but the studies did not associate the cognitive assessment and simple reaction time measures. Nine articles that met three inclusion criteria were reviewed. We found that executive function and learning and memory have been associated with complex and choice reaction time measures. Language, perceptual motor function, and attention have been studied with mixed evidence for an association with reaction time; whereas, sensation and perception and motor construction have not been assessed. Overall, limited research has compared cognitive domain function and simple reaction time to determine if age-related changes are associated. While the complex interplay between cognition and motor function is of substantial interest, these measures are often interdependent and additional knowledge is needed to understand their influence on sensorimotor control with age.

